# Andes virus reaches Europe: a narrative review on clinical challenges, diagnostic gaps and public health preparedness, 2026

**DOI:** 10.2807/1560-7917.ES.2026.31.42.2600467

**Published:** 2026-10-22

**Authors:** Giuseppe Sberna, Bernadett Pályi, Klara Sondén, Eliana Specchiarello, Aleksandra Pettke, Guilherme Dias de Melo, Silvia Meschi, Fabrizio Carletti, Chammiran Daniel, Zoltan Kis, Hervé Bourhy, Fabrizio Maggi, Johan Aarum, Jonas Klingström, Francesca Colavita

**Affiliations:** 1 Laboratory of Virology and Laboratories of Biosafety, National Institute for Infectious Diseases Lazzaro Spallanzani – IRCCS, Rome, Italy; 2 National Biosafety Laboratory, National Center for Public Health and Pharmacy (NNGYK), Budapest, Hungary; 3 European Union Public Health Reference Laboratories for Emerging, Rodent-borne and Zoonotic Viral Pathogens (EURL-PH-ERZV); 4 Public Health Agency of Sweden, Solna, Sweden; 5 Department of Medicine Solna, Karolinska Institutet, Stockholm, Sweden; 6 Institut Pasteur, Université Paris Cité, Lyssavirus Epidemiology and Neuropathology Unit, Paris, France; 7 Division of Molecular Medicine and Virology, Department of Biomedical and Clinical Sciences, Linköping University, Linköping, Sweden

**Keywords:** Andes virus, hantavirus, person-to-person transmission, diagnostics, zoonosis

## Abstract

**BACKGROUND:**

Up to 2 July 2026, a total of 13 cases of Andes virus (ANDV) including three deaths were reported in connection to an outbreak on the cruise ship m/v *Hondius*, which departed from Argentina in April and subsequently travelled to Europe to evacuate the passengers. This event prompted all European health authorities to take action to limit the spread of the virus.

**AIM:**

To synthesise current knowledge on ANDV, with particular attention on laboratory diagnosis and capability gaps.

**METHODS:**

A literature search on ANDV was conducted, with a focus on human infection, transmission, outbreak investigations, countermeasures and diagnostics. To provide context for the ship-linked outbreak, updates from official public health sources, including the World Health Organization and European Centre for Disease Prevention and Control were used. Moreover, activities by European Union Public Health Reference Laboratories for Emerging, Rodent-borne and Zoonotic Viral Pathogens (EURL-PH-ERZV) were briefly presented.

**RESULTS:**

Andes virus is unique among hantaviruses because of sustained person-to-person transmission. Diagnosis relies on integration of clinical and epidemiological information with molecular detection of viral RNA or serological evidence. At the time of the outbreak, European diagnostic preparedness was generally well established, but important gaps remained prompting rapid support from the EURL-PH-ERZV.

**CONCLUSIONS:**

The recent event demonstrated how a geographically restricted zoonotic infection can pose public health challenges beyond endemic areas. This highlights the need for strong collaboration between clinical services, public health authorities and reference laboratories to strengthen surveillance, early detection, risk assessment and diagnostic preparedness for emerging pathogens.

## Introduction

*Orthohantavirus andesense* [1,2], commonly known as Andes virus (ANDV), is one of the most prominent New World orthohantaviruses. Andes virus is mainly transmitted to humans by rodents, but unlike other orthohantaviruses, person-to-person transmission upon close contact has been documented, which poses additional challenges and difficulties for public health [3-5]. 

First discovered in Argentina in 1995 [6], ANDV has been recognised as one of the main causes of hantavirus cardiopulmonary syndrome (HCPS) [4,7]. Andes virus infection is associated with substantial lethality, with reported case fatality rates of ca 20–40% [3,8]. Andes virus is currently considered endemic in Chile and Argentina [4,9-11], although rare isolated imported cases have been documented [12]. 

In early 2026, an ANDV outbreak was detected on the Dutch-flagged expedition cruise vessel m/v *Hondius*. The cruise vessel departed from Ushuaia, Argentina, on 1 April 2026, bound for the African continent carrying a total of 147 passengers and crew from 23 countries, including nine European Union/European Economic Area (EU/EEA) countries [13-15]. The outbreak prompted all European health authorities to take action to contain and limit the spread of the virus by tracing epidemiological links and strengthening diagnostic preparedness. By 2 July 2026, according to the World Health Organization (WHO) outbreak situation report, a total of 13 cases had been reported, including 12 laboratory-confirmed infections with ANDV and one classified as a probable case [13]. The overall attack rate was 8.8% (13 individuals infected of 147 on board), and three deaths were recorded, corresponding to a case fatality rate of ca 23% [13]. A genomic epidemiological outbreak investigation revealed that the sequences share a high degree of genetic similarity, with only two synonymous mutations found in the L segment across the cluster. This indicates that the outbreak originated from a single zoonotic spillover event, followed by human-to-human transmission [16,17]. 

This narrative review provides an overview of the current knowledge on ANDV, its epidemiology, clinical features, treatment and management of confirmed/suspected cases, with a particular focus on laboratory diagnosis and capability gaps. The activities by European Union Public Health Reference Laboratories for Emerging, Rodent-borne and Zoonotic Viral pathogens were presented.

## Methods

### Eligibility criteria

Eligible publications included peer-reviewed original studies, case reports and case series, outbreak and surveillance investigations, diagnostic validation or comparative studies, clinical and observational treatment studies, relevant animal and in vitro studies, and systematic or narrative reviews. Priority was given to studies directly addressing diagnostic methods, countermeasures, biosafety issues or clinical-management questions about ANDV. Studies on other hantaviruses were included only when they provided information relevant to aspects discussed in the review. Publications were excluded when they were duplicates, lacked sufficient relevant information, or did not contribute to the clinical, epidemiological, diagnostic, therapeutic, biosafety or public health objectives of the review. No publication date restriction was applied. No language restrictions were imposed at the initial search stage.

### Search strategy

The scholarly literature was searched using PubMed, Medline and Google Scholar. Search terms combined the synonyms ‘Andes virus’, ‘Andes hantavirus’ and ‘Orthohantavirus andesense’ with terms relating to human infection, hantavirus cardiopulmonary syndrome, clinical features, transmission, person-to-person transmission, outbreaks, diagnosis, RT-PCR or nucleic acid amplification testing (NAAT), serology, treatment, countermeasures, vaccines, infection prevention and control, and biosafety. Official updates from WHO, European Centre for Disease Prevention and Control (ECDC), and relevant local public health authorities were reviewed separately to contextualise the m/v *Hondius* ship-linked outbreak.

### Study selection and data synthesis

Titles and abstracts were screened for relevance, followed by full-text assessment of key publications. Studies were selected for inclusion when they met the eligibility criteria and contributed directly to one or more of the review questions (including ANDV overview and updated data on epidemiology, transmission, clinical syndromes, diagnostics and countermeasures). The synthesis distinguished, where relevant, primary clinical evidence, observational and outbreak evidence, diagnostic method studies, preclinical evidence, case reports and review-level evidence. No formal risk-of-bias instrument was applied across all study types, and no meta-analysis was performed.

## Results

### Viral structure, entry and replication

Andes virus (species *Orthohantavirus andesense*, genus *Orthohantavirus*, family Hantaviridae, order Elliovirales) has pleomorphic enveloped virions of approximately 80–120 nm in diameter. Its negative-sense single-stranded RNA genome is about 12 kb long and comprises three segments: L, M and S, encoding the RNA-dependent RNA polymerase, the surface glycoproteins Gn and Gc, and the nucleoprotein, respectively [4]. The Gn and Gc glycoproteins mediate cell attachment and membrane fusion, while the nucleoprotein associates with viral RNA to form ribonucleoprotein complexes. Andes virus also encodes a non-structural protein (NSs), which may contribute to evasion of the host innate immune response [18]. Its segmented genome allows reassortment among closely related hantavirus variants [19]. Cell entry occurs through receptor-mediated endocytosis, followed by endosomal membrane fusion and cytoplasmic viral transcription and replication [18,20,21].

### Pathogenesis and clinical features

Upon infection in humans, ANDV primarily targets vascular endothelial cells, causing HCPS [22], although some infections may be unrecognised because of asymptomatic or mild symptoms. This suggests that not all infected individuals develop a severe form of the disease, confirmed by cross-sectional seroprevalence studies in healthy individuals [23,24]. 

Hantavirus cardiopulmonary syndrome was first described in 1993 as a highly fatal respiratory illness in an outbreak in the south-western United States, caused by a novel hantavirus later named Sin Nombre virus [25]. The first identification of ANDV-related HCPS occurred in 1995 in Argentina, demonstrating that HCPS was not limited to North America [7]. Hantaviruses primarily target the endothelial cells lining capillaries and small vessels, and the mechanism responsible for HCPS is a substantial increase in vascular permeability, resulting from functional alteration in the endothelial barrier. This dysfunction is not due to a direct cytopathic effect of the virus, but rather to an excessive host immune response [26], which leads to massive plasma leakage into the extravascular compartment [3,27]. 

The incubation period of HCPS appears to vary, perhaps due to the route and duration of exposure. For zoonotic transmission following exposure in a high-risk area for less than 48 h, the incubation period has been estimated, by an observational study, to range from 14 to 32 days; however, for longer exposure periods, up to 14 days, the reported range extends from 7 to 39 days [28]. In the context of human-to-human transmission, incubation periods ranging from 9 to 40 days have been reported [5].

Irrespective of mode of infection, the incubation period is followed by three clinical phases: prodromal, cardiopulmonary and convalescent; in more severe cases, death may result. In the prodromal phase, patients present influenza-like symptoms, i.e. high fever, muscle aches and headache. The cardiopulmonary phase, caused by increased vascular permeability, manifests with different symptoms like cough, dyspnoea, tachycardia, hypoxia and non-cardiogenic pulmonary oedema; in severe cases, cardiogenic shock, lactic acidosis and massive haemoconcentration may also occur [3,29]. The convalescent phase is characterised by the gradual resolution of pulmonary oedema and a slow but complete clinical recovery in most patients, usually requiring several months [3,29]. The reported death rate is around 20–40% [3,8]. Moreover, an asymptomatic case has been reported with a transient low-level viraemia [30]. In a recent prospective observational study, disease severity was associated with the presence of ANDV RNA in one or more body fluids, i.e. saliva, urine, gingival crevicular fluid, in addition to blood (OR: 2.58; 95% CI: 1.42–5.18) [31].

### Transmission

#### Zoonotic transmission

The long-tailed pygmy rice rat, *Oligoryzomys longicaudatus*, is recognised as the main natural ANDV reservoir, and is predominantly found in endemic areas of South America, i.e. Chile and Argentina [4]. These rodents can sustain persistent, asymptomatic infection and shed the virus through urine, faeces and saliva, leading to environmental contamination. As with other hantaviruses, the risk of spillover to humans is linked to ecological exposure in rural and peri-urban habitats. Infection usually occurs through inhalation of aerosols or dust from infected rodent excreta or contact with contaminated materials and surfaces; rodent bites are a rare transmission route [32]. Human risk is therefore strongly linked to human behaviours that increase exposure to contaminated rodent excreta, including cleaning unused buildings, farming, camping, handling firewood, entering enclosed rural structures, and occupational or recreational activities in endemic wilderness areas [33,34]. The recent ship-linked outbreak identified ‘birdwatching’ as a risk factor and a possible source of infection, supporting the theory of zoonotic transmission [35].

#### Person-to-person transmission

Andes virus exhibits a distinctive epidemiological feature compared to other hantaviruses: close contact with infected people can lead to secondary transmission. The first documented evidence of human-to-human transmission of ANDV emerged during an outbreak in 1996–1997 associated with small household and community transmission in Argentina [6,36,37]. Over the past 30 years, person-to-person transmission has been documented [5,38] in other outbreaks, including household and nosocomial transmission [39]. These studies identified several possible modes of transmission: close person-to-person contact shortly before the onset of fever, contact with contaminated clothing, and direct exposure during patient care [39]. According to a retrospective observational investigation, the largest ANDV outbreak reported was in 2018 in the Chubut Province, Argentina, located in the Patagonia region [5], with a total of 34 confirmed cases and 11 deaths. Here, a single rodent-to-human introduction triggered a sustained person-to-person chain of transmission driven by three super-spreading events linked to crowded social gatherings resulting in a median reproductive number of 2.12 [5]. Furthermore, a prospective observational study from 2024 using viral infection assays suggested that certain biological fluids such as gingival crevicular fluid and saliva contain infectious virus, reinforcing the plausibility of person-to-person spread via oral or respiratory secretions and supporting stricter infection control measures [31]. In accordance, in an in vitro study, ANDV is less susceptible to the antiviral capacity of saliva than other hantaviruses [40].

### Countermeasures

#### Infection and viral spread control

Since currently no vaccine is available, current measures for controlling ANDV infections include general public health measures. Firstly, it is essential to take precautions to limit exposure to rodent excreta, the main source of transmission to humans [32]. Secondly, if however the virus has already spread to humans, rapid and early identification of suspected cases is needed, as well as the tracing of close contacts [5,32,39]. Finally, it is essential to implement appropriate precautions in healthcare and home settings to prevent nosocomial and household infections [3,5,39]. These measures particularly address droplet and close-contact transmission given the documented viral shedding in oral and respiratory secretions [31], including not only nosocomial/domestic close contact, but also the correct handling of clothing and bed linens that has come into contact with an infected person [39,41]. In vitro studies have reported the efficacy of various surface disinfectants against ANDV, confirming the usefulness of common commercially available alcohol-based, aldehyde-based and hydrogen peroxide-based products in healthcare settings and outbreak scenarios [42]. Recently, a report on a healthcare worker not directly involved in caring for an imported ANDV case was published, showing a transient positive ANDV PCR result without subsequent symptoms or seroconversion. Although the exact transmission route could not be determined, this situation highlights that, for the occupational safety of healthcare workers, clear and trained procedures are essential in the management of patients with ANDV infection [30]. 

#### Therapeutic and prophylactic options

Currently, specific treatment options for ANDV-infected patients are lacking and supportive care remains essential [3,43,44]. The rapid progression of disease represents a major clinical challenge and may rapidly lead to severe cardiopulmonary failure. Intensive cardiopulmonary support, i.e. oxygenation, mechanical ventilation, vasoactive support and, in refractory cardiopulmonary failure, veno-arterial extracorporeal membrane oxygenation, may be required in severe cases, suggesting improved survival in patients with fulminant disease [45]. In a small observational clinical case series in 2022, two of three patients treated with VA-ECMO survived, whereas the patient cannulated during cardiac arrest died [45]; thus, VA-ECMO remains the principal rescue intervention. 

Clinical trials have not demonstrated any benefit from adjunctive therapies. In a non-randomised multicentre clinical trial, treatment of patients with HCPS with human immune plasma appeared safe but did not significantly reduce mortality [46]. High-dose methylprednisolone treatment was not associated with significant reduction in clinical deterioration or mortality in a randomised, placebo-controlled clinical trial [47]. In preclinical in vitro and animal studies, human monoclonal antibodies from ANDV survivors or patients infected with other orthohantaviruses, such as Puumala virus, showed strong neutralising activity and complete protection in hamster models when administered a few days after viral challenge but before symptom onset [47-50]. Their demonstrated role is therefore post-exposure prophylaxis or very early treatment; protection may be substantially reduced once symptomatic disease begins. The same timing caveat applies to ribavirin and favipiravir and may partly explain the discrepancy between preclinical protection and clinical results. In a double-blind, placebo-controlled ribavirin trial, survival without VA-ECMO was similar to ribavirin and placebo (70% vs 62%), with no evidence of benefit in established cardiopulmonary disease [51-53].

Other antiviral and host-directed approaches remain investigational. In a preclinical animal study, favipiravir protected hamsters when administered up to 4 days after infection, but not after viraemia began [54]. Icatibant has been associated with clinical improvement in isolated Puumala virus case reports, but this does not establish efficacy for ANDV [55,56]. In a Spanish case report linked to the m/v *Hondius* outbreak, ribavirin, favipiravir, icatibant and baricitinib were administered together; the patient recovered, but the combination treatment and early diagnosis prevent attribution of benefit to any individual drug [57]. Recently, a potential new therapeutic opportunity has emerged with tocilizumab. This IL-6 receptor antagonist was administered to five patients with severe hantavirus pulmonary syndrome, four of whom survived to intensive care unit (ICU) discharge, compared with none of five eligible patients who did not receive the drug [58]. However, the small, non-randomised design and treatment selection constraints preclude causal inference, and thus, these preliminary findings need further evaluation [58].

Finally, although no authorised vaccine is yet available, progress is being made on vaccine development [59-61]. Preclinical platforms that have been explored for development of an ANDV vaccine include DNA and recombinant subunit vaccines, vesicular stomatitis virus (VSV) and modified vaccinia Ankara (MVA) vectors, and newer mRNA platforms. Protective effects of these vaccine platforms have been reported in animal models, but the only human phase 1 evidence concerns an ANDV DNA vaccine, which was safe and induced a robust, durable immune response [59-61]. Inactivated vaccines against Hantaan and Seoul viruses are used in China and South Korea, but they target different orthohantaviruses; they are not ANDV vaccines or Western-authorised ANDV countermeasures [62,63].

### Diagnosis and biosafety aspects

#### Laboratory diagnosis consideration

The diagnosis of ANDV infection is based on a combination of clinical, epidemiological and laboratory findings. Diagnostic confirmation is achieved through direct detection of ANDV viral RNA using molecular biology techniques and/or through serological tests to demonstrate the presence of an ANDV-specific antibody response. Laboratory diagnosis is essential to confirm or exclude ANDV infection and to support timely clinical management, infection prevention and control measures, including contact tracing. It also plays a key role in outbreak investigation and surveillance by helping to monitor geographic spread and transmission patterns. Although evidence is lacking that serial testing of asymptomatic contacts of a ANDV case reduces onward transmission, serial testing may support early detection, closer clinical monitoring, infection control decisions, and the study of interventions intended to prevent disease progression. However, according to WHO and ECDC, tests performed during the incubation period may be negative and therefore do not exclude subsequent infection [64,65].

#### Laboratory biosafety measures

For laboratory risk assessment, ANDV should be treated as a Risk Group 3 pathogen and security-sensitive biological agent. According to WHO, virus propagation should always be performed under a minimum of Biosafety Level 3 (BSL-3) containment [64,66]. Inactivation of clinical specimens should preferably also be performed under BSL-3 containment. If a validated inactivation procedure is exceptionally conducted in a BSL-2 laboratory after a documented risk assessment, a Class III biosafety cabinet or other fully closed primary-containment device should be used. A Class II cabinet should be reserved for strictly limited procedures under enhanced and heightened control measures. As always, good microbiological practice and procedure (GMPP) must be applied including good general behaviours and aseptic techniques, in line with the WHO laboratory biosafety manual [66] and national provisions. To ensure appropriate handling of ANDV isolates and clinical samples from suspects/confirmed cases, each laboratory must perform its own risk assessment based on materials to be handled in combination with the procedure to be performed as well as the availability of control measures, including laboratory facilities and internal procedures in place.

#### Molecular testing

Nucleic acid amplification testing is the recommended method for early diagnosis [67]. Moreover, NAAT can detect ANDV RNA in blood specimens 5 to 15 days before the onset of symptoms or the appearance of IgM antibodies in exposed and infected individuals [41]. These methods include real-time or conventional PCR detecting specific sequences of ANDV RNA; alternatively, hantavirus-generic RT-PCR can be used targeting S and/or L segments, followed by genomic sequencing to confirm ANDV. Viral RNA can be detected in different human specimen types [31], including whole blood, plasma, urine, gingival crevicular fluid and saliva [31]. In the presence of respiratory symptoms, samples from the respiratory tract may also be indicated, as they can provide useful diagnostic support [31]. However, this type of sampling is not yet part of routine clinical practice in endemic countries and its sensitivity has been shown to be lower than that of RT-PCR on blood. Nevertheless, the presence of the virus in respiratory tract samples may reflect the widespread distribution of the virus within the host. The use of cellular fractions of blood samples, particularly white blood cells (WBC), as suitable targets for molecular detection has been demonstrated in several studies, although this specimen type may be less practical than whole blood for routine diagnostic workflows. In this context, RT-qPCR on WBC samples showed a sensitivity of 94.9% and a specificity of 100% [67]. Moreover, in a study involving more than 130 ANDV-positive patients in the acute phase of infection in Chile, WBC samples tested positive in more than 90% of cases from 0 to 23–29 days after symptom onset, i.e. throughout the acute phase and also during the convalescent phase [31]. In contrast, positivity in all other biological fluids analysed (including plasma) declined more rapidly over time [31]. Notably, in a longitudinal case report, ANDV RNA was detected for at least 71 months in the semen of a patient who was monitored over time; this led the study authors to hypothesise that the virus may have found a biological sanctuary site in which to replicate, and suggest the possibility of sexual transmission [68]. Additionally, as further support in an outbreak situation, next generation sequencing techniques [9] can detect viral mutations that might affect transmissibility, disease severity, or diagnostic performance. In addition, genomic sequencing investigation is crucial to define transmission events and cluster characterisation [16,17].

#### Serological testing

According to the United States Centers for Disease Control and Prevention (US CDC) and ECDC international guidelines and numerous studies, serological testing remains an important routine reference method for the diagnosis of orthohantaviruses [69-72]. In ANDV infection, IgM and IgG antibodies are often detectable early from symptom onset. Specifically, IgM antibodies may be detectable within 1–2 days, whereas IgG antibodies are generally detectable within 7 days after onset of symptoms [73]. IgM typically wanes within ca 3 months, while IgG persists for years. IgM is considered a marker of recent exposure, while IgG is indicative of either acute or convalescence and past infection [74]. Evidence of seroconversion or a fourfold rise in titres between paired serum samples ​with either IgM or IgG antibodies also supports confirmation of an acute ANDV infection [75]. However, cross-reactivity with other hantavirus species of the same serogroup (mainly with Sin Nombre virus, and also the Old World orthohantavirus Puumala and Dobrava-Belgrade viruses) may limit the specificity of the result. It can produce false-positive results, especially in areas where other orthohantaviruses are endemic or co-circulate, which may be the case of several European regions (such as Puumala virus in Scandinavia and other European countries, and Dobrava virus in Balkan regions). Commercially, various technologies are available: ELISA, immunoblotting, indirect immunofluorescence and rapid immuno-chromatographic tests; many of which are designed for the broader diagnosis of hantaviruses and thus not specific to ANDV [76-79].

For the ANDV serology and serotyping of orthohantaviruses, the N-protein was largely used to establish several assays, including tests based on the expression of truncated viral N-proteins in yeast or bacteria [73,80-82]. Serotyping ELISAs are useful for epidemiological surveillance in regions where two or more hantavirus species co-circulate [81]. The recombinant N-protein of Puumala hantavirus, which cross-reacts with other hantaviruses, has been used also in a rapid immunochromatographic test [80]; although this assay is not specific for ANDV, it demonstrated sensitivity and specificity above 90%, indicating good cross-reactivity to the Puumala nucleoprotein antigen. Lederer et al. developed a CE-marked indirect immunofluorescence assay on a biochip that included an ANDV substrate [78]. Although this assay achieved full sensitivity for IgG and IgM against ANDV, it also exhibited cross-reactivity with the Sin Nombre virus [78].

Overall, serological evidence supports the use of these tests as screening or confirmatory tools, while the ANDV-specific reference ELISA and, especially, early molecular tests remain more reliable benchmarks for cases with suspected acute infection. Notably, there is limited use of additional confirmatory assays such as neutralisation tests. These are further complicated by the absence of a clear cytopathic effect, requiring alternative protocols, including fluorescence-based assays [83-85].

#### Diagnostic gaps

The interpretation of results obtained from ANDV tests depends closely on the timing of the tests and on the epidemiological data, i.e. living area of the patient, recent travel to consider cross-reactivity between several hantaviruses; combining all these aspects remains therefore valuable to establish a definite diagnosis. Indeed, at the onset of symptoms or during the early prodromal phase, molecular tests on peripheral blood cells can detect the infection when serology is still negative [41,67]; however, as the disease progresses, serology becomes increasingly relevant [73]. This implies that a negative serological result in the early stages of disease does not rule out active infection.

It is clear, however, that although NAAT and serology are effective, ready-to-use field-based approaches for early diagnosis remain limited [69,70]. Rapid point-of-care approaches and lateral flow formats are still under evaluation [86,87]. The issue of cross-reactivity between hantaviruses also needs to be considered; for example, hantavirus N-protein contains both highly cross-reactive antibody targets and hantavirus species-specific epitopes [88], which could aid in the development of assays for the specific diagnosis of ANDV.

Finally, it is also important to recognise that available reports suggest that person-to-person transmission is concentrated around the early febrile or prodromal phase, with no documented transmission during the cardiopulmonary or convalescent phases [5,31]. Presymptomatic transmission remains uncertain, and the timing and duration of infectiousness require further study. This implies that outbreak response should not be based solely on late laboratory confirmation.

### Readiness of European laboratories

European Centre for Disease Prevention and Control established the European Union Reference Laboratory for Public Health (EURL-PH) network in 2015 to strengthen Europe's rapid response and resilience, promoting strong laboratory leadership and a harmonised response among network members. The EURL-PH on Emerging, Rodent-borne and Zoonotic Viral Pathogens (EURL-PH-ERZV) became operational on 1 January 2025. At the beginning of the ANDV outbreak, the EURL-PH-ERZV launched a survey about ANDV diagnosis to assess the level of technical readiness and the need for diagnostic support. The survey was sent out on 9 May 2026, and within 2 days, 29 laboratories from 24 European countries had responded. The overall results showed that 17 of 24 countries had either molecular or serological capacity to detect ANDV, four had molecular capacity only, while three countries had serological capacity only; 10 countries had already established both capacities. Of the countries having molecular capacity for ANDV or other orthohantaviruses, the majority of the countries (13/17) rely on in-house methods including conventional pan-hantaviruses PCR and/or ANDV-specific real-time RT-PCR.

Regarding serology, most methods available were not ANDV-specific and relied on cross-reactivity against other orthohantaviruses. Of those, six countries had commercial assays including ELISA, indirect immunofluorescence assays (IFA) or Western blot, while four had ANDV-specific assays.

At the time of the survey, whole genome sequencing capacity had already been implemented in 12 countries using both long- and short-reads platforms and various techniques (e.g. amplicon-based, metagenomics, etc.); all laboratories recorded capacity of bioinformatics analysis with short time-to-results (average: 1–2 days). The EURL-PH-ERZV rapidly distributed positive-control materials, provided advice on biosafety measures and protocols, supported to laboratories lacking diagnostic capacity, and offered technical support and expert advice to ECDC.

## Conclusions

Andes virus was first discovered 3 decades ago, and although its ability to infect humans is well established, our knowledge of its impact on human health remains limited. Moreover, its emergence through imported or travel-related cases raises new questions regarding preparedness in non-endemic areas such as Europe.

Currently, ANDV is not considered to have clear pandemic potential. While person-to-person transmission of ANDV has been reported, to date it has mainly been associated with close and prolonged contact. It is important to highlight that, despite the low case numbers, risk is not negligible. Rather, the relatively limited number of reported cases probably reflects the rarity of spillover from infected rodent reservoirs to humans and the relatively low basic reproductive rate of ANDV in the human population. In the European context, the possibility of direct zoonotic spillover of ANDV is reduced by the absence of natural rodent reservoirs. However, this does not exclude the potential for residual risk: the possibility of the virus being introduced via imported cases still requires a careful assessment of the human population’s susceptibility, as well as the virus’s ability to sustain human-to-human transmission chains. Episodes in closed settings such as the ship-linked cluster represent a particular opportunity of study. In this context, it is possible to observe the infection dynamics of human-to-human transmission of ANDV with greater precision, helping to clarify aspects that are not yet fully understood. Therefore, it is essential to promote targeted studies involving the entire public health system to obtain a more complete assessment of the ANDV’s potential impact in non-endemic areas like Europe.

Given the lack of available large-scale countermeasures, rapid identification of cases remains essential for disease control through case isolation and monitoring close contacts. This is particularly important in indoor settings, healthcare facilities and other contexts where close contact may occur. The cornerstone of this process is the laboratory diagnosis: it is essential for confirming cases, excluding ANDV infection and guiding prevention and control measures. Diagnostic capacity may be limited in non-endemic countries because ANDV infections are extremely rare. Key challenges include limited access to validated commercial assays, confirmatory testing, and specialised reference laboratory support. In addition, the serological cross-reactivity between the orthohantaviruses complicates interpretation of the results, together with the scarcity of reference materials and external quality assessment schemes needed to support assay validation and quality assurance. All these considerations make it clear that emerging viruses have a relevant impact and implications for preparedness in non-endemic regions such as Europe.

Strong networking between clinical services, public health authorities and national and international reference laboratories is essential to strengthen surveillance, early case detection and infection prevention and control measures in closed settings and point-of-entry settings, e.g. airports, seaports, cruise terminals, and land border crossings. Joint laboratory efforts are also needed to support the development, validation and implementation of diagnostic assays for rare or emerging pathogens such as ANDV.

## Data Availability

All data are available upon request from the authors.
